# A pilot feasibility randomised controlled trial of an adjunct brief social network intervention in opiate substitution treatment services

**DOI:** 10.1186/s12888-018-1600-7

**Published:** 2018-01-15

**Authors:** Ed Day, Alex Copello, Jennifer L. Seddon, Marilyn Christie, Deborah Bamber, Charlotte Powell, Carmel Bennett, Shabana Akhtar, Sanju George, Andrew Ball, Emma Frew, Ilias Goranitis, Nick Freemantle

**Affiliations:** 1 Birmingham and Solihull Mental Health NHS Foundation Trust, c/o Dept of Psychiatry, The Barberry 25 Vincent Drive, Edgbaston, Birmingham, B152FG UK; 2 Addictions Department, Institute of Psychiatry, Psychology & Neuroscience, King’s College London, London, UK; 3 School of Psychology, The University of Birmingham, Birmingham, UK; 4 Leicester City Drug and Alcohol Service, Leicester, UK; 5 Health Economics Unit, College of Medical and Dental Sciences, University of Birmingham, Birmingham, UK; 6 Institute of Clinical Trials and Methodology, University College London, London, UK

**Keywords:** Randomised controlled trial, Social networks, Social behaviour and network therapy (SBNT), Heroin use, Opioid substitution therapy

## Abstract

**Background:**

Approximately 3% of people receiving opioid substitution therapy (OST) in the UK manage to achieve abstinence from prescribed and illicit drugs within three years of commencing treatment. Involvement of families and wider social networks in supporting psychological treatment may be an effective strategy in facilitating recovery, and this pilot study aimed to evaluate the impact of a social network-focused intervention for patients receiving OST.

**Methods:**

A two-site, open feasibility trial randomised patients receiving OST for at least 12 months but still reporting illicit opiate use in the past 28 days to one of three treatments: 1) treatment as usual (TAU), 2) Brief Social Behaviour and Network Therapy (B-SBNT) + TAU, or 3) Personal Goal Setting (PGS) + TAU. The two active interventions consisted of 4 sessions. There were 3 aims: 1) test the feasibility of recruiting OST patients to a trial of B-SBNT, and following them up over 12 months; 2) test the feasibility of training clinicians to deliver B-SBNT; 3) test whether B-SBNT reduces heroin use 3 and 12 months after treatment, and to explore potential mediating factors. The primary outcome for aim 3 was number of days of heroin use in the past month, and a range of secondary outcome measures were specified in advance (level of drug dependence, mental health, social satisfaction, therapist rapport, treatment satisfaction, social network size and support).

**Results:**

A total of 83 participants were randomised, and 70 (84%) were followed-up at 12 months. Fidelity analysis of showed that B-SBNT sessions were clearly distinguishable from PGS and TAU sessions, suggesting it was possible to train clinical staff to an adequate level of competence.

No significant differences were found between the 3 intervention arms in the primary or secondary outcome measures. Attendance at psychosocial treatment intervention sessions was low across all three arms (44% overall).

**Conclusions:**

Patients receiving OST can be recruited into a trial of a social network-based intervention, but poor attendance at treatment sessions makes it uncertain whether an adequate dose of treatment was delivered. In order to achieve the benefits of psychosocial interventions, further work is needed to overcome poor engagement.

**Trial registration:**

ISRCTN Trial Registration Number: ISRCTN22608399.

Date of registration: 27/04/2012. Date of first randomisation: 14/08/2012.

**Electronic supplementary material:**

The online version of this article (10.1186/s12888-018-1600-7) contains supplementary material, which is available to authorized users.

## Background

The first decade of the twenty-first century saw a step change in the size and scope of treatment services for illicit drug users in England, with increased financial investment leading to a doubling of the number of people in treatment in the period 2000–2005 alone [1]. The political drive behind this change was a desire to reduce criminal activity, but with a newly-elected UK Government in 2010 came a change in direction. Since then there has been more focus on ‘recovery’, with a national strategy requiring treatment agencies to focus on increasing the number of patients leaving their service abstinent from all illicit and prescribed drugs, in good mental and physical health, and with improved quality of life [2]. Supporters of this approach noted that this goal was rarely achieved, with only 3% of people reporting abstinence within three years of starting opiate substitution treatment (OST) in one study in Scotland [3]. Treatment services often focused on medication to the exclusion of effective psychosocial interventions for the opiate dependent population [4], and one large-scale attempt to evaluate Cognitive Behavioural Therapy in OST clinics identified major training and organisational barriers [5].

The social environment can directly support and reinforce drug use, particularly if a majority of an individual’s social network is made up of other drug users [6–9]. One potential strategy to support abstinence from illicit drugs is to encourage patients of OST services to end all contact with drug-using peers. However, this is unlikely to happen if no alternative social contact or support is available, and so psychosocial treatment approaches have begun to focus on building links to individuals and groups who provide competitive reinforcement for abstinence. When this is done successfully, the development of new positive social supports is associated with a reduced risk of relapse to heroin and other drug use and better overall outcomes [10–14].

The important role of families and communities in recovery from drug use has been emphasised in recent treatment policy in England [15, 16], with the National Treatment Agency for Substance Misuse (NTA) signalling its enthusiasm for involving families and other social network members in the treatment process (pp. 15) [17]. However, despite support for change from both researchers and policy makers, drug treatment agencies tend to focus on the individual rather than the family or wider community. Further work is needed to develop and evaluate treatment interventions that aim to build social support for change in substance use and other behaviours, but also to understand the barriers to implementing such an approach. In an increasingly pressured financial environment, psychosocial interventions must be feasible in routine clinical practice and acceptable to both service users and clinicians. They must also move beyond merely targeting changes in drug use, but focus on the pursuit of the wider goal of recovery, including mental and physical health, employment and quality of life [18]).

We report the findings of a pilot trial with the aim of implementing, observing and assessing the efficacy of a social-network intervention (Brief-Social Behaviour and Network Therapy – B-SBNT) for both opiate substitution treatment patients and their social network members. This paper reports on three of the trial’s main objectives:

1. To test the feasibility of recruiting patients engaged in drug treatment services for at least a year but still reporting heroin use to a trial of B-SBNT, and following them up over 12 months.
2. To test the feasibility of training NHS clinicians with no previous experience of working with social network members to deliver a brief social network-driven intervention (B-SBNT).
3. To test whether B-SBNT reduces heroin use 3 and 12 months after treatment, and to explore potential mediating factors.

The full study evaluation also included a comprehensive economic evaluation which has been published elsewhere [19, 20].

## Method

### Design

This early phase open pilot study had a pragmatic, multicentre, randomised parallel group design, comparing a 4-session social network intervention (B-SBNT) with an individually-based personal goal setting intervention of equivalent intensity (PGS), and with treatment as usual (TAU). It targeted patients receiving OST for more than 12 months but still reporting opiate use in the preceding 28 days, and was conducted within three National Health Service (NHS) community drug treatment teams in two UK regions; the West Midlands (Solihull and Birmingham) and the East Midlands (Leicester).

### Intervention

Participants allocated to either of the active treatment groups received an intervention as an adjunct to usual care, delivered by a different therapist to the participant’s usual drug worker.

1. Treatment as usual (TAU):

In the control arm participants received usual care with no additional therapy sessions. The treatment services allowed the clinicians to utilise a therapeutic style of their choice for working with patients, and TAU sessions did not follow a treatment manual. Some drug workers had been exposed to goal setting and SBNT techniques as part of their previous training and development. However, previous evaluation in these services had shown that TAU sessions occurred between weekly and fortnightly, lasted an average of 45 min, and focussed on case management, signposting of other services, and other activities such as medication issues [21]. Therefore TAU would consist of interventions that were both less structured and less frequent than the two active treatments.

2. Brief Social Behaviour and Network Therapy (B-SBNT) ± TAU:

Social Behaviour and Network Therapy (SBNT) is built on the idea that social support for change is central to resolving substance dependence and seeks to involve close friends and family as part of the treatment process [22]. The approach was shown to be effective and cost-effective in the large UK Alcohol Treatment Trial (UKATT) [23, 24]. Subsequently, therapists from community drug services in Birmingham were trained to deliver the intervention at a two-day training workshop and then guided by a written manual and supervision using video-recordings of sessions. An evaluation concluded that it was feasible to train 12 therapists to deliver SBNT, with the participating patients reporting a reduction in drug use and improved family and social relationships at the 3-month follow-up point [25].

Originally SBNT was developed as an 8-session intervention, although in reality 64% of the UKATT sample (*n* = 320) received no more than 4 sessions [26]. Some of the most important components occur during the early part of the treatment e.g. drawing a social network diagram; contacting and inviting people; reviewing communication and interactions with significant network members. The original intervention manual [25] was therefore adapted for the current study using a four session format, and is hereafter referred to as Brief Social Behaviour and Network Therapy (B-SBNT). The most effective components of the SBNT intervention [26] were combined with elements of node-link mapping [27] to facilitate the training and delivery of the intervention. The therapist worked with the patient to draw a ‘social network diagram’ during the first session in order to identify potential social support for change that could be drawn upon in later sessions [28]. Network members identified by the participant were then approached and invited to take part in treatment sessions, and the therapist used elements of communication skill development, coping behaviours and the development of joint activities to support the development of a network-supported relapse management plan. B-SBNT was delivered by a trained clinician that wasn’t the patient’s usual key worker. Participants were invited to attend four 50-min B-SBNT sessions over a period of 6 weeks, in addition to TAU sessions with their usual keyworker. They were asked to nominate at least one ‘network’ member who would then be actively encouraged to participate in subsequent sessions.

3. Personal Goal Setting (PGS) ± TAU:

PGS involved an additional active component to usual care through a structured process of setting personal goals and monitoring their attainment. This arm was included to control for the intensity of treatment in the B-SBNT arm and the process of receiving the intervention from a different therapist. PGS consisted of four 50-min sessions over a period of 6 weeks delivered according to a purpose designed manual [29] by a trained clinician. It was based on the principles of node-link mapping [27] and included a review of the participant’s current situation and future aspirations, the development of SMART (Specific, Measurable, Agreed-upon, Realistic, Time-limited) goals, and monitoring and feedback on progress in achieving these goals.

### Treatment monitoring and fidelity

To ensure that B-SBNT and PGS were delivered with sufficient fidelity, all trial therapists were required to participate in monthly supervision meetings with research clinicians. Each therapeutic session with the therapist was audio recorded, with a proportion randomly selected for fidelity assessment using the UKATT Process Rating Scale [30].

### Staff recruitment & training

As the aim of the study was to test the feasibility of training staff to deliver a social network intervention, no attempt was made to randomly select therapists and clinicians volunteered to participate. They were trained using the process adopted in previous SBNT pilot work [25], with an initial one-day training session to introduce the key concepts and procedures involved in each treatment intervention. All staff delivering B-SBNT were then required to pilot the methods with one clinical case prior to the commencement of the trial. Monthly 90-min group supervision sessions were provided for both active treatment conditions, and clinicians brought node-link maps developed in the session to guide discussion.

### Participant recruitment & randomisation

#### Inclusion and exclusion criteria

Each potential participant had to (1) be 18 years or older; (2) have been prescribed OST (methadone or buprenorphine) continuously for the past 12 months; (3) report use of heroin on one or more days in the previous 28 days; (4) give informed consent to participation. The only reason for exclusion was a severe co-morbid mental or physical health issue that prevented an individual from participating in treatment sessions. Any such cases were discussed with clinicians at the time of screening.

The Treatment Outcome Profile (TOP) assessment is completed every 3–6 months for all patients receiving specialist substance misuse treatment in England [31], and includes questions about use of illicit opiates. These data are stored as part of the patient’s electronic record unless the patient has refused consent to share this information, and so the research team were able to identify individuals that met the study inclusion criteria by reviewing the case record every 6 weeks. A list of potential participants was given to clinical staff in each participating team, and the patient was given an initial overview and written information about the study at their next routine appointment with their usual clinician. If the patient expressed an interest they were contacted by a researcher who provided further information and invited them to sign a consent form. Patients were randomised and allocated to treatment intervention following completion of the baseline interview. A dynamic randomisation algorithm was used, minimizing differences in the numbers allocated to each experimental group [32]. As this was an open trial, randomisation was not stratified by investigational site as this would not be properly concealed, and was done using a secure, remote randomisation service independent of the research team.

### Assessments

Assessment was conducted before treatment randomisation (baseline), and at 3 and 12 months post-baseline. Sociodemographic data was only collected at baseline, and included age, sex, ethnicity, employment status, and living situation. Ten other instruments were administered at all three time points, and further details are given in the study protocol [33].

(i) Illicit drug use: section B of the Maudsley Addiction Profile (MAP) [34] was used to quantify the number of days on which heroin, cocaine, benzodiazepine or alcohol was used in the previous 28 days, and the average amount of use of each drug on each using day. A urine sample was taken to test for heroin, methadone, buprenorphine, cocaine and benzodiazepines.
(ii) Health risk behaviour: section C of the MAP [34] to quantify the number of days on which drugs were injected in the previous 28 days.
(iii) Drug-related problems: section E of the MAP [34] was used to quantify the number of days of work and acquisitive crime in the previous 28 days.
(iv) Severity of drug dependence: the Leeds Dependence Questionnaire (LDQ) [35] is validated in drug users and is composed of 10 questions that are summed to give a maximum score of 30. A higher score indicates greater dependence.
(v) Psychological functioning: measured using three domains of the Clinical Outcome in Routine Evaluation (CORE-OM) scale [36] (a) well-being (4 items); (b) symptoms (12 items: 4 depression, 4 anxiety, 2 trauma, 2 physical); (c) functioning (12 items – 4 general functioning, 4 functioning in social relationships, 4 functioning in close relationships). Each item is scored between 0 and 4, and a mean score is reported for each domains.
(vi) Social functioning: the Social Satisfaction Questionnaire (SSQ) [37] is an 8-item self-complete questionnaire that measures perceived social problems. The maximum score is 24, and higher scores represent greater satisfaction with housing, finances or relationships.
(vii) Motivation to change behaviour: the Readiness to Change Questionnaire–Treatment Version (RTCQ-TV) [38] is a 15-item questionnaire that allows the assignment of substance users to one of the stages described in Prochaska and DiClemente’s model of change (i.e. Precontemplation, Contemplation or Action) [39].
(viii) Social network structure and function: the Important People Drug & Alcohol Interview (IPDA) [14] is a researcher-administered instrument that requires respondents to provide the first name and relationship of up to 10 members of their social network who have been important to them in the previous three months. For each network member identified the respondent then rates their frequency of contact, importance, general support, frequency of use of drugs or alcohol, and their reaction to the participant’s substance use and treatment.
(ix) General social support: the Interpersonal Support Evaluation List (ISEL) [40] is a self-completion instrument that requires the participant to rate 40 statements about the availability of possible social resources. Four 10-item subscales are then generated, measuring ‘Tangible’ support, ‘Appraisal’ support, ‘Self-esteem’ support, and ‘Belonging’ support. Each subscale is scored between 0 and 30, where a higher score indicates a greater perception of support.
(x) Therapeutic engagement: the 36 items that make up the Engagement section of the Client Evaluation of Self and Treatment (CEST) [41] were completed by the participant, with each item scored between 1 and 5. This generated 4 sub-scale scores representing ‘Treatment Participation’, ‘Treatment Satisfaction’, ‘Counselling Rapport’, and ‘Peer Support’.

### Sample size

As this was a pilot feasibility study, a formal sample size calculation was not appropriate. However, by using a recruitment goal of 120 participants (i.e. 40 in each treatment arm), we calculated that if the proportion of patients that stopped taking heroin in the B-SBNT group was 0.3 (e.g. 30%), this would produce an approximate 95% confidence interval of 0.18–0.44 (e.g. 18% to 44%) for this estimate. Alternatively, if the proportion was found to be 0.1 (e.g. 10%), an approximate 95% confidence interval would be 0.01–0.19 (e.g. 1% to 19%).

### Data analysis

#### Trial outcome analysis

Data was analysed according to the intention-to-treat principle; all randomised participants were included in the analysis irrespective of whether they stayed in the trial or not, with missing data treated as failing to achieve the outcome. The major analyses were pre-specified in a statistical analysis plan completed prior to database lock. Analyses were conducted in SAS 9.2 or above (www.sas.com/software/sas9). The primary outcome measure was the number of days that the participant had used heroin in the previous 28 days. The primary analyses compared B-SBNT with the other two arms (PGS and TAU), and subjects were analysed using a generalised mixed model with Gaussian mixed error structures, including experimental group as an explanatory classification variable. The therapists were included as random effects [42]. Analysis of continuous secondary outcomes was conducted using analogous statistical models, including changes in level of injecting drug use, criminal activity, psychological symptoms, severity of drug dependence, motivation for drug abstinence, social satisfaction, therapeutic engagement, social network structure and function, and general social support.

### Session rating

Each audio-recorded treatment session was rated by the two independent raters (CB and SA) using a standardised measure of fidelity for SBNT developed for the UK Alcohol Treatment Trial (UKATT) [30]. Inter-rater reliability was assessed using a two-way mixed, consistency, average-measures Intra Class Correlations (ICC) [43] to assess the degree that coders provided consistency in their ratings of the frequency and quality items across sessions.

## Results

### Sample

**Fig. 1:**
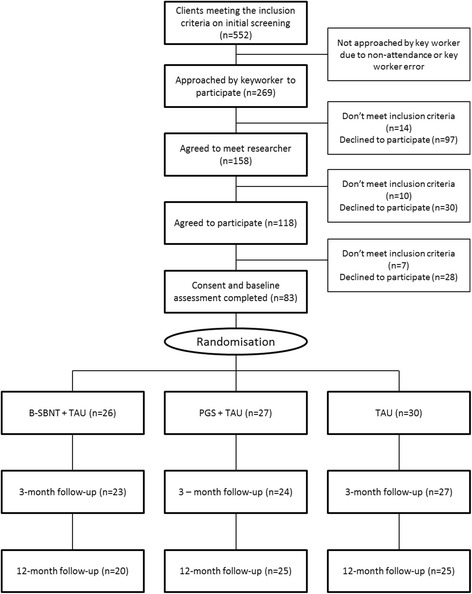
Flow diagram for the trial, consistent with the Consolidated Standards of Reporting Trials (CONSORT) 2010 statement

The study began in NHS services in the metropolitan areas of Solihull and Leicester. However, the service in Leicester underwent a re-commissioning process early in the trial period which led to a large reorganisation that drew resources away from the study. Staff were uncertain of their jobs throughout the trial period, and the disruption to clinical services hampered screening and recruitment. As a result, the Leicester site managed only 32% of its target of 60 participants, whereas Solihull recruited 90%. A further site (Birmingham) was added to compensate for this shortfall, but the overall recruitment level fell short of the target level at a total of 83 opiate dependent patients. Figure 1 shows a flow diagram for the trial, consistent with the Consolidated Standards of Reporting Trials (CONSORT) 2010 statement [44].

Participants were randomly allocated to SBNT+TAU (*n* = 26), PGS + TAU (*n* = 27) or TAU only (*n* = 30). The primary outcome was illicit opiate use, measured by urine screen and participant self-report. In addition, participants completed secondary outcome measures at baseline (*n* = 83), 3-months (*n* = 74) and 12-months (*n* = 70) post-treatment.

### Study aims

*1. To test the feasibility of recruiting patients engaged in drug treatment services for at least a year but still reporting heroin use to a trial of B-SBNT, and follow them up over 12 months.*

The mean age of the sample was 37 years (range 25–61), and 72 (87%) participants were male. The participants were predominantly white British (*n* = 69, 83%), unmarried (*n* = 75, 90%), and unemployed (*n* = 63, 76%). Seventeen (21%) had been receiving OST for between 12 and 24 months, 40 (48%) between 25 and 60 months, and 25 (30%) for over 5 years. Most (*n* = 51, 61%) expressed a goal of being abstinent from all opiates (prescribed and illicit) within 12 months. Seventy six of the participants (82%) were prescribed methadone solution and 7 (18%) buprenorphine sublingual tablets. The median dose of methadone was 60 mg/day (range 14 to 106), and the median dose of buprenorphine was 8 mg (range 6 to 16 mg).

### Trial engagement & participation

A total of 1524 patients were treated in the 3 services during the period of study, and just over a third (552, 36%) were prescribed OST and reported opiate use in the past month according to the latest TOP. The protocol required the patient’s regular key worker to approach patients meeting the inclusion criteria to enquire about their interest in participating in the study. Just under half (269, 49%) were approached, of which 118 (21%) were willing/able to discuss entering the trial, and 83 (15%) consented to participate. Reasons for refusal included not wishing to participate in research, not wanting extra treatment sessions, or having other commitments such as work or family that prevented regular attendance.

### Research follow-up

Once recruited to the study, the retention rate was good (84% at 12-months), and the system of tracing participants by accessing their patient records, contacting their GP or pharmacist, or liaising with their drug worker was effective.

*2. To test the feasibility of training NHS clinicians with no previous experience of working with social network members to deliver a brief social network-driven intervention (B-SBNT).*

### Training

Clinicians were asked to volunteer to be trial therapists, and 16 were trained in the 2 intervention arms (7 B-SBNT, 9 PGS). The attendance rate at the monthly group supervision sessions was 66%, and clinicians presented cases using node-link maps generated in sessions and discussed specific techniques. There was a wide variation in the number of study cases taken on by each trained therapist, but SBNT therapists had a median of 2 cases (range 1 to 12), PGS therapists a median of 2 cases (range 1 to 10), and TAU therapists a median of 1 case (range 1 to 4).

#### Participant engagement

There was a high level of non-attendance at treatment sessions, with 49% of the planned sessions completed in the B-SBNT group, 27% in the PGS group and 50% in the TAU group. Therapists varied in their ability to engage participants in treatment within each trial arm.

### Session recordings and fidelity ratings

Overall, participants attended 115 treatment sessions (47 BSBNT, 29 PGS, 39 TAU). Sixty one session recordings were available for analysis (26 BSBNT, 20 PGS, 4 TAU). The sessions lasted between 19 and 67 min (M = 34.4 min; see Additional file 1 for session duration by study arm). Therapy sessions were rated on items measuring Session Management (maintaining structure, agenda setting, explanation of treatment, reviewing inter-session change, consistency of problem focus, and end of sessions summary), Specific Tasks (homework, alternatives to drinking/drug use, social support for change-general, involvement of others in behaviour change and identifying support for change), and Therapist Style (therapist as task oriented, therapist as active agent for change, collaboration, interpersonal focus). Each of these items was rated on frequency and quality on Likert scales ranging from 0 to 4. Additionally, raters recorded the type of treatment maps that were completed during sessions. Additional file 2 shows the average ratings for item frequency, quality and number of maps used per session by study arm. Both raters gave higher frequency and quality ratings for B-SBNT session than for PGS or TAU sessions. A greater number of maps were completed in PGS sessions than in B-SBNT sessions, while a greater number of maps was completed in both active study arms compared to TAU.

Each session was rated by the two independent raters. Inter-rater reliability was assessed using a two-way mixed, consistency, average-measures Intra Class Correlations (ICC; McGraw & Wong, 1996) to assess the degree that coders provided consistency in their ratings of the frequency and quality items across sessions. The resulting ICC’s ranged from .43 to .95 and .41 to .93 for quality and frequency respectively (see Additional file 3), indicating that coders had a high degree of agreement for the majority of variables and suggesting that the majority of variables were rated similarly across coders (poor consistency for the item consistency of problem focus only).

*3. To test whether B-SBNT reduces heroin use 3 and 12 months after treatment, and to explore potential mediating factors.*

There were no significant changes in the primary outcome (combined self-report + objective drug use) between B-SBNT +TAU, GS + TAU, and TAU only at 3-month and 12-month follow-up (see Table 1). Likewise, as Table 2 shows, there were no significant differences between the three groups in any of the secondary outcome measures at either 3-months or 12-months.

**Table 1:** Differences in the primary outcome measure between the three trial arms

	Median days abstinent from heroin				
	Baseline	3 months	12 months	Relative risk (95% CI) vs TAU 3 months	Relative risk (95% CI) vs TAU 12 months
B-SBNT	15	18	17	0.96 (0.79–1.17)	0.96 (0.78–1.17)
PGS	18	18	18	0.93 (0.77–1.13)	1.00 (0.83–1.20)
TAU	20	23	25

**Table 2:** Differences in the secondary outcome measure between the three trial arms

	SBNT (*n* = 26)			PGS (*n* = 27)			TAU (*n* = 30)		
Item	Baseline	3 months	12 months	Baseline	3 months	12 months	Baseline	3 months	12 months
Days abstinent from heroin, median (IQR)	15 (8,24)	18 (2,22)	17 (11,23)	18 (8,24)	18 (8,20)	18 (8,27)	20 (12,26)	23 (14,26)	25 (14,27)
LDQ total, median (IQR)	14 (8,17)	9 (5,16)	10 (6.6,16)	11 (5,15)	6 (5,12)	7 (4,10)	7 (2,13)	6 (3,12)	5 (2,13)
CORE									
Wellbeing, median (IQR)	2 (2,3)	2 (0.8,2.5)	2 (1.3,2.9)	2 (1,2)	1.6 (0.8,2.1)	1.5 (0.8,2)	2 (1,2)	1.3 (0.8,2)	1.5 (0.8,2.3)
Symptoms, median (IQR)	2 (2,3)	2.1 (0.9,2.9)	2.4 (1.1,2.6)	2 (1,2)	1.3 (1,2.3)	1.5 (0.8,2)	2 (1,3)	1.3 (1,2.3)	1.6 (0.8,2.3)
Functioning, median (IQR)	2 (2,3)	1.8 (1.2,2.6)	2.2 (1,2.7)	2 (1,2)	1.5 (1.1,2)	1.6 (0.8,2)	2 (1,2)	1.5 (1.1,2.1)	1.2 (0.8,1.9)
SSQ total, median (IQR)	23 (19,26)	24 (22,28)	24 (19.5,27)	22 (18,25)	23 (19,24)	22 (20,26)	23 (20,27)	24 (20,28)	24 (22,27)
CEST									
Counselling rapport, median (IQR)	40 (38,43)	41 (38,43)	39 (35,46)	39 (36,42)	39 (38,42)	40 (39,42)	40 (39,43)	40 (39,44)	41 (40,48)
Peer support, median (IQR)	32 (28,40)	30 (26,38)	30 (25,37)	30 (28,34)	30 (28,32)	30 (26,34)	32 (28,38)	32 (26,36)	36 (28,38)
Treatment participation, median (IQR)	38 (33,39)	38 (36,39)	38 (31,42)	37 (35,38)	37 (35,38)	38 (35,40)	38 (34,41)	39 (37,42)	41 (40,47)
Treatment satisfaction, median (IQR)	39 (35,41)	40 (36,43)	40 (35,40)	39 (37,41)	39 (37,41)	40 (36,42)	40 (39,43)	40 (39,44)	41 (40,47)
ISEL									
Appraisal, median (IQR)	16 (15,19)	17 (15,18)	18 (14,21)	16 (14,18)	18 (16,20)	18 (16,20)	16 (15,18)	18 (17,20)	18 (16,20)
Belonging, median (IQR)	17 (14,19)	16 (14,18)	17 (14,19.5)	15 (13,17)	17 (15,19.5)	16 (14.5,19.5)	17 (15,18)	17 (16,20)	18 (15,21.5)
Self-esteem, median (IQR)	14 (12,17)	14 (13,15)	13.5 (12,16.5)	14 (12,16)	15 (14,17)	15 (14,18)	16 (13,18)	15 (14,18)	15 (14,20)
Tangible, median (IQR)	17 (13,19)	16 (14,19)	16 (14,19.5)	15 (14,19)	16 (15,19)	18 (14.5,19.5)	16 (14,18)	16 (14,17)	17.5 (15.5,21)
IPDA									
Index 1-network size (IQR)	−0.42 (−0.66,0.24)	0.07 (−0.83,0.83)	−0.29 (−0.74,1.01)	− 0.18 (− 0.66,0.64)	−0.14 (− 0.83,0.65)	0.12 (− 0.74,0.49)	0.24 (− 0.18,1.0)	0.07 (− 0.83,1.18)	0.12 (− 0.29,0.84)
Index 2–daily network size (IQR)	− 0.50 (−1.06,0.06)	− 0.10 (− 0.58,0.37)	−0.49 (− 0.76,0.06)	−0.06 (− 0.50,1.18)	−0.10 (− 0.58,0.37)	−0.49 (− 0.49,0.61)	−0.22 (− 0.50,0.06)	−0.50 (− 0.50,0.62)	0.06 (− 0.49,0.61)
Index 3-importance (IQR)	−0.14 (− 0.65,0.62)	0.17 (− 0.33,0.75)	−0.12 (− 0.58,0.67)	−0.02 (− 1.08,1.25)	−0.01 (− 0.89,0.94)	−0.12 (− 0.62,0.38)	−0.02 (− 0.86,1.25)	−0.01 (− 0.43,0.75)	0.38 (− 0.12,1.14)
Component 1: substance involvement (IQR)	−0.40 (− 0.93,0.05)	−0.35 (− 0.83,0.62)	−0.28 (− 0.79,1.14)	−0.06 (− 0.44,0.67)	−0.24 (− 0.51,0.34)	−0.37 (− 0.84,0.16)	−0.24 (− 0.75,0.85)	−0.49 (− 0.66,0.47)	−0.07 (− 0.58,0.57)
Component 2: general/treatment support (IQR)	0.14 (− 0.56,0.66)	−0.04 (− 0.48,0.57)	0.24 (− 0.58,0.66)	−0.49 (− 1.03, 0.30)	−0.10 (− 0.50,0.74)	−0.14 (− 0.83,0.43)	0.48 (− 0.35,1.02)	0.09 (− 0.17,0.77)	0.34 (− 0.30,0.82)
Component 3-support for substance use (IQR)	0.16 (− 0.86,0.89)	0.25 (− 0.75,0.82)	0.48 (− 0.72,1.37)	0.34 (− 0.86,0.89)	0.08 (−0.75,0.68)	− 0.51 (− 0.94,0.90)	0.02 (− 0.86,0.72)	0.07 (− 1.02,0.57)	−0.51 (− 0.94,0.68)
RCQ-TV, median (IQR)	4.5 (4,7)	5 (4,6)	4 (4,7)	4 (3,5)	4 (3,5)	4 (4,6)	4.5 (4,6)	4 (4,6)	5 (4,7)

The mean number of appointments offered differed significantly between the three arms of the study (B-SBNT+TAU = 6.2, PGS + TAU = 4, TAU = 2, F = 30.11, *p* < 0.001), but the mean rates of attendance were not significantly different (B-SBNT+TAU = 54%, PGS + TAU = 49%, TAU = 63%). During the 6-week study period participants received a mean of 1.8 B-SBNT and 1.1 PGS sessions, in addition to a mean of 1.3 TAU sessions, representing an attendance rate of 44% overall. Sixteen participants (19.2%) attended no sessions at all during the study period. Eleven (42%) of the 26 participants randomised to B-SBNT brought a network member to a session (7 for 1 session, 1 for 2 sessions and 3 for 3 sessions), compared with none in the PGS and one in the TAU groups.

As the focus of the intervention was on changing social support for recovery from opiate dependence, the IPDA data are explored in more detail. For the whole sample, the median number of people considered important with whom they had contact in the preceding 90 days was 5 (range 2–10). Family members were the most frequently named group (median = 3.2, range 0–10), followed by friends (1.4, 0–6), members of the recovery community (0.5, 0–4), and co-workers (0.2, 0–4). Nearly 80% (66/83) could name 4 ‘very important people’, and none of this sub-group accepted or encouraged drug use. A clear majority of the reported network (84%) supported the participant attending drug treatment services. Heavy drug users made up just 7% of the reported network, and a median of 4 out of 5 network members named were totally abstinent from illicit drugs.

## Discussion

The National Institute for Health and Care Excellence (NICE) has highlighted the limits of the evidence base for psychosocial treatments for heroin dependence, but also noted the promise of family and social network interventions [45]. Here an attempt was made to specifically adapt the SBNT intervention to make it more suitable for use within drug treatment services by using the elements found to be most important in the process of change [26]. Although it was possible to train clinical staff to an adequate level of competence (fidelity analysis of the audiotapes showed that B-SBNT sessions were clearly distinguishable from the PGS and TAU sessions), and to follow-up a high proportion (84%) of study participants for 12 months, the study highlights two major challenges to implementation of this feasibility study in UK treatment services.

The first relates to organisational barriers to conducting research with this population in OST services in England. The current national policy of re-tendering the contract to provide treatment services [46, 47] means that the service provider may change every three years. Not only is this disruptive to patients and staff, but it also presents a significant impediment to conducting clinical research. One site in this trial was unaffected by re-commissioning during the trial period and recruited 90% of its target in the available time; the other underwent a re-commissioning process three months after the study started and recruited a third of its target. The need to compete to win a new contract possibly drew vital resources away from the clinical ‘front line’ and distracted otherwise motivated clinicians from their research role. As substance use disorders have come to be understood as chronic illnesses that require a chronic disease management model, so continuity of treatment provision becomes more important [48]. In a review of the evidence base for Recovery Oriented Systems of Care, White highlights stability in terms of funding, organizational ownership and workforce is a key measure of quality [49].

The second challenge concerns the potential participants’ reluctance to engage in psychosocial therapy. The population under study was the most intractable group in treatment i.e. opiate dependent individuals who had received OST at flexible dosing levels for at least one year but had not stopped using heroin. Many had been receiving OST at a therapeutic dose for at least 5 years and were still reporting heroin use on more than half of the preceding 28 days, in addition to regular use of crack cocaine. Across the three sites 552 patients met the eligibility criteria, but only 83 (15%) started the study. Overall 155 (28%) patients refused the offer of participating when they were initially assessed or failed to attend the assessment appointment. This reflects the difficulty of making contact with patients of UK OST services, but also their reluctance to receive interventions in addition to an OST prescription. Less than two-thirds of the sample (51/83) saw their goal of treatment to be abstinent from both illicit (i.e. heroin) and prescribed (i.e. OST) opiates in the next year, with the rest either unsure of their goal (*n* = 9) or aiming for abstinence from illicit opiates only (*n* = 23). Nearly 20% of the participants did not attend any treatment sessions during the study period, although there was some evidence once engaged the social network intervention was more likely to be completed (8 (30.7%) B-SBNT cases completed the full 4-session program compared to 2 (7.4%) PGS participants).

The benefits of OST in this population are well documented [50], but the effect of additional psychosocial treatment is not clear [4]. There is a body of evidence that shows that adding more service produces better outcomes [51–53], but when specific psychosocial interventions are added the evidence base is not as strong [54]. This study found no difference in any measure between the three treatment arms. This mirrors recent findings in prescription opioid dependence where the addition of counselling to OST made no difference to drug related outcomes [55], and in treatment-refractory opioid dependent individuals where there was no evidence for the superiority of a cognitive behavioural intervention over an active comparison condition [56]. Another study cited loss of hope as a reason for non-attendance at counselling sessions [57]. This was a feasibility study of a promising intervention, and the next planned step was to move to a larger, adequately powered trial. However, for this study to lead to a definitive trial it would have needed to provide an indication of positive change in the main outcome measure for the social network intervention. Incomplete recruitment meant a lack of power to determine potentially small effects, and it is also likely that the ‘dose’ of social network treatment administered was inadequate to change behaviour in a group with such complex and enduring needs. During the 6-week study period participants attended less than 50% of the treatment sessions offered to them (active intervention or routine appointments), and less than half of the B-SBNT cases involved a social network member in a treatment session.

Orford has called for a shift in the way addiction research is conducted, with less emphasis on studying named interventions and more focus on studying change processes within the broader, longer-acting systems of which treatment is part [58]. Previous research has shown that some patients benefit from OST without the need for additional non-medical treatment, whereas others only benefit when an adequate dose of psychosocial intervention is added [53, 59]. Rates of abstinence-based recovery are low in this population, and McKay has argued that treatments need to link patients to reinforcers that will make continued abstinence more appealing [60]. Interventions that extend beyond the individual to the family and wider social network are a good way of doing this. However, in order to increase the amount of exposure to therapeutic components it may be necessary to incorporate ‘evidence based practices’ (EBPs) into routine care [61], rather than trying to deliver a whole new additional intervention package. A greater emphasis on finding suitable reinforcers for participation may be needed, and contingency management strategies have shown promise in this population [62, 63]. The variability in therapist involvement in this trial also reminds us that there should also be a greater focus on studying therapist factors in existing treatment staff, and future attempts might start by integrating social network-orientated thinking into a whole team approach.

## Conclusions

This study has shown that promising interventions to harness social network support for change are feasible in the OST population, and staff can be trained to deliver them. However, further work is required to tailor the intervention to this population, possibly through the addition of more potent reinforcers to attend therapy sessions. It has also highlighted the need for a stable workforce and organizational structure within which to deliver elements of a chronic care model.

## Additional files

Additional file 1: Minimum, maximum, and mean session duration in minutes by trial arm. A comparison of the treatment session lengths between the three trial arms. (DOCX 16 kb)

Additional file 2: Mean ratings for item frequency, quality and number of maps used per session by study arm. Each audio-recorded treatment session was rated by two independent raters using a standardised measure of fidelity for SBNT. This file presents data from this measure, as well as information about the number of node-link maps produced in each session. (DOCX 14 kb)

Additional file 3: Inter-rater reliability for frequency and quality ratings. Each session was rated by the two independent raters, and inter-rater reliability was assessed using Intra Class Correlations to assess the degree that coders provided consistency in their ratings of the frequency and quality items across sessions. (DOCX 16 kb)

## Abbreviations

B-SBNT: Brief Social Behaviour and Network Therapy
CEST: Client Evaluation of Self and Treatment
CONSORT: Consolidated standards of reporting trials
CORE-OM: Clinical outcome in routine evaluation – outcome measure
EBP: Evidence-based practice
ICC: Intra class correlation
IPDA: Important people drugs and alcohol
IQR: Inter-quartile range
ISEL: Interpersonal support evaluation list
LDQ: Leeds dependence questionnaire
MAP: Maudsley addiction profile
NHS: National Health Service
NICE: National Institute for Health and Care Excellence
NTA: National treatment agency
OST: Opioid substitution therapy
PGS: Personal goal setting
RTCQ-TV: Readiness to change questionnaire – treatment version
SBNT: Social behaviour and network therapy
SMART: Specific, measurable, agreed-upon, realistic, time-limited
SSQ: Social satisfaction questionnaire
TAU: Treatment as usual
TOP: Treatment outcome profile
UKATT: UK alcohol treatment trial

## References

[1] Best D, George S, Day E, Day E (2007). The development of the drug treatment system in England. Clinical topics in addiction.

[2] HM Government (2010). Drug strategy 2010. Reducing demand, restricting supply, building recovery: supporting people to live a drug free life.

[3] McKeganey N, Bloor M, Robertson M, Neale J, MacDougall J (2006). Abstinence and drug abuse treatment: results from the drug outcome research in Scotland study. Drugs Educ Prev Policy.

[4] Day E, Mitcheson L (2017). Psychosocial interventions in OST services: does the evidence provide a case for optimism or nihilism?. Addiction.

[5] Drummond DC, Kouimtsidis C, Reynolds M, Russell I, Godfrey C, McCusker M, Coulton S, Parrott S, Davis P, Tarrier N et al: The effectiveness and cost effectiveness of cognitive behaviour therapy for opiate misusers in methadone maintenance treatment: a multicentre randomised controlled trial (UKCBTMM). Final report to the Department of Health Research and Development Directorate. In. London: Department of Health; 2004.

[6] Best D, Hernando R, Gossop M, Sidwell C, Strang J (2003). Getting by with a little help from your friends, the impact of peer networks on criminality in a cohort of treatment-seeking drug users. Addict Behav.

[7] Gogineni A, Stein M, Friedmann PD (2001). Social relationships and intravenous drug use among methadone maintenance patients. Drug Alcohol Depend.

[8] Latkin C, Mandell W, Oziemkowska M, Celentano D, Vlahov D, Ensminger M, Knowlton A (1995). Using social network analysis to study patterns of drug use among urban drug users at high risk for HIV/AIDS. Drug Alcohol Depend.

[9] Schroeder JR, Latkin CA, Hoover DR, Curry AD, Knowlton AR, Celentano DD (2001). Illicit drug use in one’s social network and in one's neighbourhood predicts individual heroin and cocaine use. Ann Epidemiol.

[10] Cohen S, Lichtenstein E (1990). Partner Behaviours that support quitting smoking. J Consult Clin Psychol.

[11] Goehl L, Nunes E, Quitkin F, Hilton I (1993). Social networks and methadone treatment outcome: the costs and benefits of social ties. Am J Drug Alcohol Abuse.

[12] Havassy BE, Wasserman DA, Hall SM (1995). Social relationships and abstinence from cocaine in an American treatment sample. Addiction.

[13] Wasserman DA, Stewart AL, Delucchi KL (2001). Social support and abstinence from opiates and cocaine during opioid maintenance treatment. Drug Alcohol Depend.

[14] Zywiak WH, Neighbors CJ, Martin RA, Johnson JE, Eaton CA, Rohsenow DJ (2009). The important people drug and alcohol interview: psychometric properties, predictive validity, and implications for treatment. J Subst Abus Treat.

[15] Centre for Social Justice (2010). Green paper on criminal justice and addiction.

[16] National Treatment Agency for Substance Misuse (2008). NTA policy on involvement of users and family members.

[17] National Treatment Agency for Substance Misuse (2010). NTA business plan 2010–11.

[18] The Betty Ford Institute Consensus Panel (2007). What is recovery? A working definition from the Betty ford institute. J Subst Abus Treat.

[19] Goranitis I, Coast J, Day E, Copello A, Freemantle N, Frew E (2017). Maximizing health or sufficient capability in economic evaluation? A methodological experiment of treatment for drug addiction. Med Decis Mak.

[20] Goranitis I, Coast J, Day E, Copello A, Freemantle N, Seddon J, Bennett C, Frew E (2016). Measuring health and broader well-being benefits in the context of opiate dependence: the psychometric performance of the ICECAP-A and the EQ-5D-5L. Value Health.

[21] Best D, Day E, Morgan B, Oza T, Copello A, Gossop M (2009). What treatment means in practice: an analysis of the delivery of evidence-based structured interventions in criminal justice drug treatment services in Birmingham, England. Addict Res Theory.

[22] Copello A, Orford J, Hodgson R, Tober G, Barrett C (2002). Social behaviour and network therapy - basic principles and early experiences. Addict Behav.

[23] UKATT Research Team (2005). Effectiveness of treatment for alcohol problems: findings of the randomised UK alcohol treatment trial (UKATT). BMJ.

[24] UKATT Research Team (2005). Cost effectiveness of treatment for alcohol problems: findings of the randomised UK alcohol treatment trial (UKATT). BMJ.

[25] Copello A, Williamson E, Orford J, Day E (2006). Implementing and evaluating social behaviour and network therapy in drug treatment practice in the UK: a feasibility study. Addict Behav.

[26] Dale V, Coulton S, Godfrey C, Copello A, Hodgson R, Heather N, Orford J, Raistrick D, Slegg G, Tober G (2011). Exploring treatment attendance and its relationship to outcome in a randomized controlled trial of treatment for alcohol problems: secondary analysis of the UK alcohol treatment trial (UKATT). Alcohol Alcohol.

[27] Dansereau DF, Simpson DD (2009). A picture is worth a thousand words: the case for graphic representations. Prof Psychol.

[28] Day E, Buckingham SA, Best D (2017). Building bridges to positive social identities: the social network diagram and opiate substitution treatment. Addiction, Behavioural change and social identity.

[29] Day E, Best D, Bartholomew NG, Dansereau DF, Simpson DD (2008). The BTEI care planning manual: mapping achievable goals. Routes to recovery.

[30] Tober G, Clyne W, Finnegan O, Farrin A, Russell I, UKATT Research Team (2008). Validation of a scale for rating the delivery of psycho-social treatments for alcohol dependence and misuse: the UKATT process rating scale (PRS). Alcohol Alcohol.

[31] Marsden J, Farrell M, Bradbury C, Dale-Perera A, Eastwood B, Roxborough M, Taylor S (2008). Development of the treatment outcomes profile. Addiction.

[32] Pocock SJ (1983). Clinical trials: a practical approach.

[33] Day E, Copello A, Seddon J, Christie M, Bamber D, Powell C, George S, Ball A, Frew E, Freemantle N (2013). Pilot study of a social network intervention for heroin users in opiate substitution treatment: study protocol for a randomized controlled trial. Trials.

[34] Marsden J, Gossop M, Stewart D, Best D, Farrell M, Strang J (1998). The Maudsley addiction profile: development and user manual.

[35] Raistrick D, Bradshaw J, Tober G, Weiner J, Allison J, Healey C (1994). Development of the Leeds dependence questionnaire (LDQ): a questionnaire to measure alcohol and opiate dependence in the context of a treatment evaluation package. Addiction.

[36] Evans C, Connell J, Barkham M, Margison F, McGrath G, Mellor-Clark J, Audin K (2002). Towards a standardized brief outcome measure: psychometric properties and utility of the CORE-OM. Br J Psychiatry.

[37] Raistrick D, Tober G, Heather N, Clark JA (2007). Validation of the social satisfaction questionnaire for outcome evaluation in substance use disorders. Psychiatr Bull.

[38] Heather N, Luce A, Peck D, Dunbar B, James I (1999). Development of a treatment version of the readiness to change questionnaire. Addict Res.

[39] Prochaska JO, DiClemente CC (1982). Transtheoretical therapy: toward a more integrative model of change. Psychother Theory Res Pract Train.

[40] Cohen S, Mermelstein R, Kamarck T, Hoberman HM, Sarason IG, Sarason BR (1985). Measuring the functional components of social support. Measuring the functional components of social support.

[41] Joe GW, Broome KM, Rowan-Szal GA, Simpson DD (2002). Measuring patient attributes and engagement in treatment. J Subst Abus Treat.

[42] SAS Institute Inc. (2009). Chapter 6: introduction to mixed Modelling procedures. SAS/STAT 92 User’s guide (second edition).

[43] KO MG, Wong SP (1996). Forming inferences about some intraclass correlation coefficients. Psychol Methods.

[44] Schulz KF, Altman DG, Moher D, Group ftC (2010). CONSORT 2010 statement: updated guidelines for reporting parallel group randomised trials. BMJ.

[45] National Collaborating Centre for Mental Health (2008). Drug misuse: psychosocial interventions.

[46] Drummond C (2017). Cuts to addiction services are a false economy. The bmj opinion.

[47] Mohammadi D (2014). Addiction Services in England: in need of an intervention. Lancet Psychiatry.

[48] AT ML, Starrels JL, Tai B, Gordon AJ, Brown R, Ghitza U, Gourevitch M, Stein J, Oros M, Horton T (2013). Can substance use disorders be managed using the chronic care model? Review and recommendations from a NIDA consensus group. Public Health Rev.

[49] White WL (2008). Recovery management and recovery-oriented systems of care: scientific rationale and promising practices.

[50] Connock M, Juarez-Garcia A, Jowett S, Frew E, Liu Z, Fry-Smith A, Day E, Linzeris N, Roberts T, Burls A (2006). Methadone and Buprenorphine for the Management of Opioid Dependence: a systematic review and economic evaluation. Health Technol Assess.

[51] Ball JC, Ross A (1991). The effectiveness of methadone maintenance treatment: patients, progress, services and outcomes.

[52] Kraft MK, Rothbard AB, Hadley TR, AT ML, Asch DA (1997). Are supplementary services provided during methadone maintenance really cost-effective?. Am J Psychiatr.

[53] AT ML, Hagan TA, Levine M, Gould F, Meyers K, Bencivengo M, Durell J (1998). Supplemental social services improve outcomes in public addiction treatment. Addiction.

[54] Amato L, Minozzi S, Davoli M, Vecchi S (2011). Psychosocial combined with agonist maintenance treatments versus agonist maintenance treatments alone for treatment of opioid dependence. Coch Database Syst Rev.

[55] Weiss RD, Potter JS, Fiellin DA, Byrne M, Connery HS, Dickinson W, Gardin J, Griffin LM, Gourevitch MN, Haller DL (2011). Adjunctive counseling during brief and extended Buprenorphine-Naloxone treatment for prescription Opioid dependence: a 2-phase randomized controlled trial. Arch Gen Psychiatry.

[56] Otto MW, Hearon BA, RK MH, Calkins AW, Pratt E, Murray HW, Safren SA, Pollack MH (2014). A randomized, controlled trial of the efficacy of an interoceptive exposure-based CBT for treatment-refractory outpatients with opioid dependence. J Psychoactive Drugs.

[57] Darker C, Sweeney B, El Hassan H, Kelly A, O’Connor S, Smyth B, Barry J (2012). Non-attendance at counselling therapy in cocaine-using methadone-maintained patients: lessons learnt from an abandoned randomised controlled trial. Ir J Med Sci.

[58] Orford J (2008). Asking the right questions in the right way: the need for a shift in research on psychological treatments for addiction. Addiction.

[59] Schwartz RP, Kelly SM, O’Grady KE, Gandhi D, Jaffe JH (2012). Randomized trial of standard methadone treatment compared to initiating methadone without counseling: 12-month findings. Addiction.

[60] McKay JR (2017). Making the hard work of recovery more attractive for those with substance use disorders. Addiction.

[61] Manuel JK, Hagedorn HJ, Finney JW (2011). Implementing evidence-based psychosocial treatment in specialty substance use disorder care. Psychol Addict Behav.

[62] Weaver T, Metrebian N, Hellier J, Pilling S, Charles V, Little N, Poovendran D, Mitcheson L, Ryan F, Bowden-Jones O, et al. Use of contingency management incentives to improve completion of hepatitis B vaccination in people undergoing treatment for heroin dependence: a cluster randomised trial. Lancet. 2015;384(9938):153-63. 10.1016/S0140-6736(14)60196-3 24725468

[63] Lussier JP, Heil SH, Mongeon JA, Badger GJ, Higgins ST (2006). A meta-analysis of voucher-based reinforcement therapy for substance use disorders. Addiction.

