# Supplementary material for: A pilot feasibility randomised controlled trial of an adjunct brief social network intervention in opiate substitution treatment services
Source: BMC Psychiatry. 2018 Jan 15;18:8. doi: 10.1186/s12888-018-1600-7 (PMC5769270; doi:10.1186/s12888-018-1600-7)
Supplement: Supplementary file 1 — Minimum, maximum, and mean session duration in minutes by trial arm. A comparison of the treatment session lengths between the three trial arms. (DOCX 16 kb) [file 12888_2018_1600_MOESM1_ESM.docx]

**ADDITIONAL FILE 1**

| **Study arm** | **Duration Type** | **Minutes** |
| --- | --- | --- |
| SBNT | Average | 32.8 |
|  | Minimum | 19.0 |
|  | Maximum | 67.1 |
| PGS | Average | 38.1 |
|  | Minimum | 20.2 |
|  | Maximum | 67.1 |
| TAU | Average | 32.3 |
|  | Minimum | 28.5 |
|  | Maximum | 37.2 |

**Minimum, maximum and average session duration in minutes by trial arm**
