# Supplementary material for: A pilot feasibility randomised controlled trial of an adjunct brief social network intervention in opiate substitution treatment services
Source: BMC Psychiatry. 2018 Jan 15;18:8. doi: 10.1186/s12888-018-1600-7 (PMC5769270; doi:10.1186/s12888-018-1600-7)
Supplement: Supplementary file 2 — Mean ratings for item frequency, quality and number of maps used per session by study arm. Each audio-recorded treatment session was rated by two independent raters using a standardised measure of fidelity for SBNT. This file presents data from this measure, as well as information about the number of node-link maps produced in each session. (DOCX 14 kb) [file 12888_2018_1600_MOESM2_ESM.docx]

**ADDITIONAL FILE 2**

| **Study arm** | **Score Type** | **Rater 1** | **Rater 2** |
| --- | --- | --- | --- |
| SBNT | Frequency | 33.4 | 34.4 |
|  | Quality | 34.2 | 35.5 |
|  | Maps | 1.2 | 1.0 |
| PGS | Frequency | 16.9 | 18.0 |
|  | Quality | 18.1 | 18.2 |
|  | Maps | 2.1 | 2.1 |
| TAU | Frequency | 17.4 | 20.8 |
|  | Quality | 18.8 | 21.2 |
|  | Maps | 0.2 | 0.2 |

**Mean ratings for item frequency, quality and number of maps used per session by study arm**
