# Supplementary material for: A pilot feasibility randomised controlled trial of an adjunct brief social network intervention in opiate substitution treatment services
Source: BMC Psychiatry. 2018 Jan 15;18:8. doi: 10.1186/s12888-018-1600-7 (PMC5769270; doi:10.1186/s12888-018-1600-7)
Supplement: Supplementary file 3 — Inter-rater reliability for frequency and quality ratings. Each session was rated by the two independent raters, and inter-rater reliability was assessed using Intra Class Correlations to assess the degree that coders provided consistency in their ratings of the frequency and quality items across sessions. (DOCX 16 kb) [file 12888_2018_1600_MOESM3_ESM.docx]

**ADDITIONAL FILE 3**

| **Items [Taken from Tober et al Ref 30]** | **ICC's Frequency** | **ICC's Quality** |
| --- | --- | --- |
| Maintaining Structure | 0.69 | 0.71 |
| Agenda Setting | 0.83 | 0.85 |
| Explanation of Philosophy of Treatment | 0.83 | 0.83 |
| Reviewing Inter - Session Change | 0.9 | 0.9 |
| Consistency of Problem Focus | 0.43 | 0.41 |
| End of Session Summary | 0.95 | 0.94 |
| Homework | 0.78 | 0.79 |
| Alternative Activities to Drinking | 0.77 | 0.76 |
| Social Support for Change - General | 0.92 | 0.89 |
| Involvement of Others in Behaviour Change | 0.93 | 0.91 |
| Identify Sources of Support for Change | 0.89 | 0.86 |
| Therapist as Task Oriented | 0.8 | 0.84 |
| Therapist as Active Agent for Change | 0.95 | 0.93 |
| Collaboration | 0.9 | 0.9 |
| Interpersonal Focus | 0.8 | 0.8 |
| Average ICC | 0.8 | 0.8 |

**Inter-rater reliability for frequency and quality ratings**
